# Evaluation and comparison of mammalian subcellular localization prediction methods

**DOI:** 10.1186/1471-2105-7-S5-S3

**Published:** 2006-12-18

**Authors:** Josefine Sprenger, J Lynn Fink, Rohan D Teasdale

**Affiliations:** 1 ARC Special Research Centre for Functional and Applied Genomics; Institute for Molecular Bioscience, University of Queensland, Brisbane, QLD 4072, Australia; 2 ARC Centre in Bioinformatics; Institute for Molecular Bioscience, University of Queensland, Brisbane, QLD 4072, Australia

## Abstract

**Background:**

Determination of the subcellular location of a protein is essential to understanding its biochemical function. This information can provide insight into the function of hypothetical or novel proteins. These data are difficult to obtain experimentally but have become especially important since many whole genome sequencing projects have been finished and many resulting protein sequences are still lacking detailed functional information. In order to address this paucity of data, many computational prediction methods have been developed. However, these methods have varying levels of accuracy and perform differently based on the sequences that are presented to the underlying algorithm. It is therefore useful to compare these methods and monitor their performance.

**Results:**

In order to perform a comprehensive survey of prediction methods, we selected only methods that accepted large batches of protein sequences, were publicly available, and were able to predict localization to at least nine of the major subcellular locations (*nucleus, cytosol, mitochondrion, extracellular region, plasma membrane, Golgi apparatus, endoplasmic reticulum (ER), peroxisome*, and *lysosome*). The selected methods were CELLO, MultiLoc, Proteome Analyst, pTarget and WoLF PSORT. These methods were evaluated using 3763 mouse proteins from SwissProt that represent the source of the training sets used in development of the individual methods. In addition, an independent evaluation set of 2145 mouse proteins from LOCATE with a bias towards the subcellular localization underrepresented in SwissProt was used. The sensitivity and specificity were calculated for each method and compared to a theoretical value based on what might be observed by random chance.

**Conclusion:**

No individual method had a sufficient level of sensitivity across both evaluation sets that would enable reliable application to hypothetical proteins. All methods showed lower performance on the LOCATE dataset and variable performance on individual subcellular localizations was observed. Proteins localized to the secretory pathway were the most difficult to predict, while nuclear and extracellular proteins were predicted with the highest sensitivity.

## Background

During the last decade a number of different methods have been generated for the computational prediction of the subcellular localization of eukaryotic proteins. Automated approaches such as these have become necessary as large-scale genomic and proteomic sequencing efforts have contributed a massive amount of protein sequence data. Automatically annotating these sequences with as much functional and structural data as possible is an important task because laboratory experiments with this same aim can be both time consuming and costly; computational predictions can thus guide future experimental efforts aimed at further investigating unknown protein sequences. In this study we evaluate how well currently available subcellular localization methods perform on two independent datasets and the extent of their coverage of subcellular compartments.

Many prediction methods have been created using manifold computational approaches with quite different results. As Dönnes *et al*. [1] mentions, publicly available methods differ mainly in four aspects: the underlying biological motivation; the computational method used; the localization coverage; and the reliability. The ultimate goal of this study was to provide guidelines on how to present novel protein sequences to a computational prediction method with the most accurate and biologically meaningful results.

## Results and Discussion

### Prediction methods

We selected five prediction methods to compare based on the following criteria: public availability, either as downloadable software or as a web server; the ability to accept large batches of sequences; and the ability to predict at least all of the nine locations: *nucleus, cytosol, mitochondrion, extracellular region, plasma membrane, Golgi apparatus, endoplasmic reticulum (ER), peroxisome*, and *lysosome*. The selected methods are CELLO [2], MultiLoc [3], Proteome Analyst [4,5], pTarget [6] and WoLF PSORT [7].

### Datasets

The LOCATE database [8,9] contains data describing the membrane organization and subcellular localization of proteins from the FANTOM3 Isoform Protein Sequence set [10], a high-quality version of the mouse transcriptome. We have performed this evaluation with proteins originating from mouse only to avoid the impact of protein orthologs within the evaluation. The localization data include primary experimental subcellular localization data, localization data mined from the literature, and localization data extracted from other databases such as UniProt/SwissProt (SwissProt Release 47) [11]. We selected proteins from the LOCATE database that were annotated with literature-mined and SwissProt-derived localization data because these sets have a relatively high coverage of the nine locations we chose to evaluate. Because it was not always possible to determine to which protein isoform the literature data referred within LOCATE, we assigned the localization term(s) to all protein isoforms encoded by the corresponding annotated gene.

In order to adhere to the controlled vocabulary, we ignored annotations representing locations that are excluded from this particular study. 2145 proteins with literature-mined annotation were selected and these are referred to as the LOC2145 dataset. 3763 proteins with SwissProt annotation were selected and these are referred to as the SP3763 dataset. 39% of the proteins within LOC2145 overlap with those in SP3763. Using the method developed by Hobohm *et al*. we determined the pairwise identities in our two data sets [12]. LOC2145 has less redundancy, when compared to SP3763, with 33% of the proteins having an identity of greater than 25% to one other protein in the set, when compared to 41% for SP3763.

The distribution of the number of locations differs markedly between the SP3763 and LOC2145 datasets and some individual proteins have multiple subcellular locations reported. Two-thirds of the SP3763 dataset represents *extracellular region *and *nucleus*, while the organelles of the secretory pathway are quite under-represented (Table 1). This under-representation of proteins in the secretory pathway, which includes the *Golgi apparatus*, *endoplasmic reticulum*, *plasma membrane*, *lysosome *and *peroxisome*, presents a particular challenge to prediction methods since there was a dearth of data on which they were trained. To overcome this issue a focused effort to identify literature reporting the localization of proteins to these under-represented locations was undertaken [9]). Therefore, the LOC2145 dataset represents a better dataset to evaluate the performance of subcellular localization predictors for these individual locations. It remains unknown if the proportion of proteins from individual subcellular localization represented in these sets truly reflects the situation *in vivo*.

### Evaluation of subcellular localization predictors

The output of some methods includes multiple predictions that are ranked by a confidence measure (such as probability or a reliability index). In such cases where the predictions were ranked it is only viable to evaluate the highest-ranked prediction. If more than one location was predicted with the same rank, we randomly selected a location for evaluation. Subcellular location predictions were generated for each protein isoform generated from an individual gene and evaluation was performed using the predicted subcellular location of the protein isoform with the highest confidence score.

Baldi *et al*. [13] defined sensitivity as the probability of correctly predicting a positive example, and the specificity as the probability that a positive prediction is correct. We used sensitivity, also known as accuracy, as the measure with which to evaluate the selected prediction methods, although this measure does not consider false positives. The sensitivity provides a convenient measure for predictive performance, but the number of proteins for various localizations is unbalanced. Therefore, we applied the specificity.

The overall sensitivity represents the proportion of correctly predicted locations not considering the individual location. With the SP3763 dataset, all five tools had an overall sensitivity in the range between 0.49 and 0.77 (Table 2). It should be noted that WoLF PSORT and Proteome Analyst trained their algorithms using BLAST [14] for homology search against the SwissProt database. Both methods achieve highest sensitivities on the dataset originating from SwissProt data with 0.61 and 0.77 respectively. CELLO's hybrid method incorporates a homology search method using the program ALIGN [15] against a dataset originating from SwissProt and its overall sensitivity was 0.58. pTarget and MultiLoc, which do not apply any homology search, had the lowest overall sensitivity of 0.49 and 0.51 respectively. The subcellular localization data within the LOC2145 dataset was derived without inclusion of information from other sources, including SwissProt, and therefore represents a suitable independent evaluation set that will have less overlap with the training sets originally used to develop the subcellular localization predictors. All of the subcellular localization predictors showed a lower level of sensitivity when applied to the LOC2145 dataset. The predictors that incorporate homology searches on SwissProt showed the largest decrease in overall performance. A similar overall sensitivity for four methods (WoLF PSORT 0.43; MultiLoc 0.43; CELLO 0.44; pTarget 0.45) was observed while Proteome Analyst displayed a higher sensitivity of 0.56. However Proteome Analyst benefited from a step in its applied method that excludes negative results. Proteome Analyst excluded 475 predictions during the test on LOC2145 (22% of the whole test set) due to low confidence. Considering these localizations as false negative predicted localizations, Proteome Analyst would have an overall sensitivity of 0.43, which is equal to the sensitivities of the other methods tested on LOC2145. Alternatively if we looked at the absolute numbers of true positive predictions then for LOC2145 Proteome Analyst was 927 that was equivalent to the other predictions which was 936 for CELLO, 913 for MultiLoc, 974 pTarget and 923 for WoLF PSORT.

In Table 2 the sensitivity and specificity for all five methods were determined for the nine individual subcellular localizations. These statistics are compared to the sensitivity and specificity expected from a random prediction. The probability for correctly predicting a location if a location is assigned at random is 0.11 for every location (sensitivity). The probability that a positive prediction is correct is equal to the proportion of the particular location in the test data set (specificity) (see Table 1). For comparison of the performance relative to individual subcellular localization we selected the dataset that contains the largest representation of that location, namely SP3763 for *nucleus*, *cytosol *and *mitochondrion *and LOC2145 for *plasma membrane*, *Golgi apparatus*, *endoplasmic reticulum*, *peroxisome *and *lysosome*. The exception was *extracellular region *for which we use LOC2145. During this analysis we observed that within SP3763 *extracellular region *35.6% of proteins were potential transmembrane proteins rather than soluble proteins free in the extracellular space. This error in nomenclature needs to be addressed when considering using subcellular localization data from Swiss-Prot for method development and evaluation [[4], unpublished observation].

Analysis of the performance of the individual predictors revealed that CELLO, MultiLoc and WoLF PSORT all displayed a clear reduction in sensitivity for the under-represented locations. Proteome Analyst generally showed the highest sensitivity for the under-represented locations but an exception was plasma membrane that had a sensitivity of only 0.16. The reason for this isolated underperformance is unknown. A number of procedural differences exist between the methods that will influence this comparison. The CELLO web server does not distinguish between plant and non-plant organisms. Therefore, animal proteins can be predicted to localize to a *chloroplast*. Proteome Analyst does not provide a prediction when the confidence in the localization is too low or no features could be extracted and used by their classifier. This reduces the number of false negatives and false positives. Because these numbers are used for the calculation of the sensitivity and specificity, they result in better and more confident results, however the coverage of the predictor is reduced. For comparison (Table 2) the achieved sensitivity for the predictions with Proteome Analyst, when all unpredicted subcellular locations are regarded as false negatives are calculated.

### Individual sensitivity and specificity per location

#### Nucleus

Proteins localized to the *nucleus *have the highest sensitivity and specificity by all five methods. For the most part, the sensitivity and specificity are mostly quite balanced, such that is there is a low number of false negatives and a low number of false positives. The most sensitive method for this location is Proteome Analyst (0.87), which also is the most specific method with 0.91.

#### Cytosol

While Proteome Analyst achieves the highest sensitivities (0.57), the majority of methods underperformed for this location. While the methods predict *cytosolic *proteins with higher values than proportional expected the levels of false negative and false positive predictions lead to low results.

#### Mitochondrion

The *mitochondrial* subcellular location is one of the most accurately predicted compartments, where all five methods achieve high sensitivity and specificity. The most sensitive method, Proteome Analyst, is also the method with the lowest number of false positive predictions.

#### Extracellular region

Location assignments to the *extracellular region *can be predicted with high sensitivity and specificity. High specificity is observed in all five methods with the highest sensitivity achieved by Proteome Analyst, but this method also tended to overpredict. The lowest sensitivity was observed in pTarget and MultiLoc, neither of which used homology search in training.

#### Plasma membrane

The majority of *plasma membrane *proteins have at least one transmembrane helix, a feature that plays a significant role in their computational localization prediction. The highest specificity achieved was by MultiLoc with a value of 0.76. It should be emphasized that the methods, MultiLoc and pTarget, which do not use homology searching both had higher than average sensitivity and specificity for this location.

#### Lysosomes, Golgi apparatus and endoplasmic reticulum

*Lysosomes, endoplasmic reticulum *and *Golgi apparatus *protein sequences are unsurprisingly poorly predicted with CELLO and WoLF PSORT failing to achieve a sensitivity above theoretical minimums for each of these classes. The sensitivity of Proteome Analyst for the *endoplasmic reticulum *and *Golgi apparatus was *0.61 and 0.58, respectively. In contrast, the sensitivity achieved for *lysosomes *was below or near theoretical minimums for all methods.

#### Peroxisome

Despite the low number of *peroxisome*-localized proteins in the test sets this localization can be well predicted by pTarget and MultiLoc.

## Conclusion

Our motivation for performing this comparison was to determine if the current subcellular localization predictors publicly available are mature enough to be productively applied to whole proteome datasets. For such an application the methods need to consider only the amino acid sequence and to predict the major subcellular locations within the mammalian cell. Many available subcellular localization methods, not considered in this comparison, require knowledge of additional protein properties including if a protein is soluble [16,17] or membrane proteins [18]. From the five methods evaluated Proteome Analyst displayed the best performance based on sensitivity and specificity. However, to what degree Proteome Analyst outperforms the other methods is difficult to estimate, as this is the only method that did not generate output for all proteins submitted. If you considered just the absolute numbers of true positives then Proteome Analyst showed an equivalent performance to the other predictors. However, all methods exhibited strengths and weaknesses on different subcellular locations and we would currently not recommend any one method above another. The highly variable performances of these datasets underscores the need for more work to be done on computational prediction methods in order to increase the accuracy, reliability, and coverage of predictive methods. Also, research targeted at elucidating more proteins that localize to compartments that are under-represented in the test datasets is crucial for the predictive methods to be trained effectively. Computational approaches for prediction of subcellular localization have variable performances, and it is important to look at the confidence (reliability index) of predictions. Excluding low prediction results does lead to a better performance and more confidence in the results, which is important for the user. Currently proteins localized to the *nucleus*, *mitochondrion *and *extracellular region *subcellular locations can be predicted with acceptable accuracy. However, it remains a challenge to develop improved prediction methods, especially for the organelles of the secretory pathway, such as *ER, Golgi apparatus*, and *lysosome*. Improved methods are required because current methods perform very poorly on these locations by generating a high number of false negatives. We believe that it is important to test these methods on independent datasets (e.g., literature-mined) because most methods are trained on SwissProt data and frequently keep highly similar sequences (i.e. protein orthologs) in the training datasets (up to 95% [6]), which then leads to overestimation of accuracy. Currently, the organelles with a larger representation in the training and test datasets (*mitochondrion, cytosol, extracellular region*, and *nucleus*) are predominantly non-membrane spanning proteins and are better predicted. Improvement in prediction of the subcellular localization of transmembrane proteins located throughout the secretory pathway clearly needs to be achieved.

We have applied these five subcellular localization methods to the mouse proteome generated from the RIKEN Functional Annotation of Mouse 3 project [10] and the output is available within the LOCATE database [8,9]. Preliminary results revealed that for proteins of unknown subcellular localization the predictors predominantly do not agree. Therefore the current methods do not appear to be relying on the same property or feature of any individual protein for their prediction.

## Methods

### Subcellular locations

We selected nine locations to which the majority of proteins are targeted. These are *nucleus, cytosol, mitochondrion, extracellular region, plasma membrane, Golgi apparatus, endoplasmic reticulum, peroxisome *and *lysosome*. These particular terms were chosen because they correspond to Gene Ontology *cellular component *terms [19]. These terms comprise a controlled vocabulary that was used throughout our study to facilitate an automated approach and comparison between methods.

### Computational subcellular localization predictors

CELLO [20] is a two-level support vector machine (SVM) classifier system based on distinctive sets of feature vectors that have been generated from primary sequence data. The dataset used to train CELLO, the PK-dataset derived from SwissProt [11], was initially described by Park *et al*. in 2003 [21]. The parameters we used for our comparison were 'Eukaryotes' as organism and 'Protein' as type of sequence.

pTARGET [22] predicts subcellular localization using amino acid composition in combination with location-specific PFAM domains [23]. This method is specialized for eukaryotic proteins and was trained on protein sequences from SwissProt (Release 45.0). It was run using all default settings.

Proteome Analyst [24] is a machine-learning method based on BLAST-inferred homology and extraction of SwissProt features. For our study with Proteome Analyst 2.5 we selected 'Animal' as the organism type.

Horton *et al*. [7] developed WoLF PSORT [25] by extending the previously-established tool PSORT. WoLF PSORT classification is based on feature selection, including amino acid composition, sequence length, and PSORT/iPSORT features. The algorithm is an adaptation of the k-nearest neighbours algorithm and also includes BLAST for homology inference. The training data set used was revised and filtered from SwissProt (Release 45.0). We selected 'Animal' as the organism type. We used the command line package version 0.1.

MultiLoc [26] is another SVM-based approach which integrates N-terminal targeting sequences, sequence motifs, and amino acid composition. MultiLoc was also trained on a dataset derived from SwissProt (Release 42.0). We used the 'MultiLoc (Animal), 9 Location' prediction method with the default parameters.

### Validation

Prediction accuracy of a method was calculated as the overall sensitivity (Equation 1).



Sensitivity and specificity for an individual location were calculated using Equation 2 and Equation 3, respectively.





where TP is the number of true positives, FN is the number of false negatives, FP is the number of false positive predicted locations and *i *is the individual location.

## Authors' contributions

JS performed the method evaluation and prepared the datasets. JLF and RDT conceptualized the project.

## Figures and Tables

**Table 1:** The distribution of individual subcellular locations in the SP3763 and LOC2145 datasets

Subcellular location	SP3763		LOC2145	
*nucleus*	1147	(26.8%)	559	(25.5%)
*cytosol*	637	(14.9%)	87	(4.0%)
*mitochondrion*	347	(8.1%)	175	(8.0%)
*extracellular region*	1547	(36.1%)	206	(9.4%)
*plasma membrane*	396	(9.2%)	703	(32.1%)
*Golgi apparatus*	96	(2.2%)	155	(7.1%)
*endoplasmic reticulum*	75	(1.7%)	163	(7.4%)
*peroxisome*	20	(0.5%)	57	(2.6%)
*lysosome*	21	(0.5%)	86	(3.9%)

Some proteins have been reported to localize to multiple subcellular locations and are thus represented multiple times in the table.

**Table 2:** Evaluation of subcellular prediction methods on SP3763 (A) and LOC2145 (B)

**A) SP3763**	**CELLO**		**MultiLoc**		**Proteome Analyst**		**pTarget**		**WoLFPSORT**		**Theoretical**	
	*Sens*	*Spec*	*Sens*	*Spec*	*Sens*	*Spec*	*Sens*	*Spec*	*Sens*	*Spec*	*Sens*	*Spec*
	
*overall*	0.58		0.51		0.77 (0.65)		0.49		0.61			
*nucleus*	0.83	0.63	0.55	0.79	0.87 (0.80)	0.91	0.62	0.78	0.75	0.74	0.11	0.30
*cytosol*	0.27	0.40	0.51	0.39	0.57 (0.49)	0.56	0.41	0.40	0.40	0.41	0.11	0.17
*mitochondrion*	0.45	0.51	0.41	0.53	0.66 (0.55)	0.81	0.50	0.50	0.45	0.41	0.11	0.09
*extracellular region*	0.47	0.82	0.41	0.81	0.70 (0.58)	0.90	0.30	0.88	0.62	0.84	0.11	0.41
*plasma membrane*	0.38	0.28	0.38	0.26	0.10 (0.07)	0.38	0.42	0.33	0.45	0.36	0.11	0.11
*Golgi apparatus*	0.04	0.80	0.08	0.08	0.48 (0.41)	0.45	0.24	0.12	0.02	0.33	0.11	0.03
*endoplasmic reticulum*	0.03	0.22	0.16	0.08	0.57 (0.43)	0.19	0.32	0.11	0.05	0.05	0.11	0.02
*peroxisome*	0.30	0.67	0.55	0.07	0.46 (0.30)	0.55	0.55	0.08	0.05	0.03	0.11	0.01
*lysosome*	0.24	0.10	0.24	0.05	0.50 (0.43)	0.19	0.19	0.02	0	0	0.11	0.01

**B) LOC2145**												

*overall*	0.44		0.43		0.56 (0.43)		0.45		0.43			
*nucleus*	0.62	0.55	0.36	0.70	0.67 (0.57)	0.92	0.42	0.71	0.49	0.71	0.11	0.26
*cytosol*	0.23	0.08	0.39	0.08	0.63 (0.51)	0.18	0.34	0.10	0.32	0.07	0.11	0.04
*mitochondrion*	0.60	0.48	0.54	0.57	0.83 (0.77)	0.80	0.59	0.43	0.52	0.43	0.11	0.08
*extracellular region*	0.65	0.30	0.58	0.33	0.92 (0.90)	0.34	0.52	0.52	0.84	0.33	0.11	0.10
*plasma membrane*	0.44	0.57	0.53	0.76	0.16 (0.10)	0.76	0.51	0.74	0.54	0.65	0.11	0.33
*Golgi apparatus*	0.05	0.78	0.25	0.44	0.58 (0.50)	0.73	0.30	0.33	0.03	1.00	0.11	0.07
endoplasmic reticulum	0.03	0.83	0.14	0.27	0.61 (0.48)	0.56	0.31	0.35	0.07	0.22	0.11	0.08
*peroxisome*	0.12	1.00	0.46	0.17	0.31 (0.20)	0.92	0.51	0.24	0.04	0.06	0.11	0.03
*lysosome*	0.05	0.25	0.09	0.11	0.17 (0.13)	0.50	0.17	0.09	0	0.06	0.11	0.04

The individual sensitivity (Sens) and specificity (Spec) achieved by each predictor overall and individual locations was calculated. Values when all unpredicted subcellular locations are regarded as failed (i.e. false negative) are shown in parentheses. When calculating the overall sensitivity, a true positive was assigned for proteins with multiple subcellular locations when any of the locations were correctly predicted.
